# Efficacy of a loading dose of IV salbutamol in children with severe acute asthma admitted to a PICU: a randomized controlled trial

**DOI:** 10.1007/s00431-022-04576-8

**Published:** 2022-08-03

**Authors:** Shelley A. Boeschoten, Corinne M. P. Buysse, Brenda C. M. de Winter, Joost van Rosmalen, Johan C. de Jongste, Rogier C. de Jonge, Sabien G. J. Heisterkamp, Job B. van Woensel, Martin C. J. Kneyber, Annelies van Zwol, Annemie L. M. Boehmer, Matthijs de Hoog, on behalf of the Dutch collaborative PICU research network (SKIC)

**Affiliations:** 1 Department of Pediatric Surgery & Intensive Care, Erasmus MC – Sophia Children’s Hospital, University Medical Center Rotterdam, Rotterdam, the Netherlands; 2 Department of Hospital Pharmacy, Erasmus University Medical Center, Rotterdam, the Netherlands; 3 Department of Biostatistics, Erasmus University Medical Center, Rotterdam, the Netherlands; 4 Department of Pediatric Pulmonology and Allergology, Erasmus MC – Sophia Children’s Hospital, University Medical Center Rotterdam, Rotterdam, the Netherlands; 5 Pediatric Intensive Care Unit, Emma’s Children’s Hospital, Amsterdam University Medical Centers, Amsterdam, the Netherlands; 6 Pediatric Intensive Care Unit, Beatrix Children’s Hospital, University Medical Center Groningen, Groningen, the Netherlands; 7 Pediatric Intensive Care Unit, Radboud University Medical Center NL, Nijmegen, the Netherlands; 8 Department of Pediatrics, Maasstad Hospital, Rotterdam, the Netherlands; 9 Department of Pediatrics, Spaarnegasthuis Hospital, Haarlem, the Netherlands; 10 Department of Epidemiology, Erasmus University Medical Center, Rotterdam, the Netherlands

**Keywords:** Intensive care, Status asthmaticus, Severe acute asthma, Therapy, Children, IV salbutamol bolus

## Abstract

The optimal dose regimen for intravenous (IV) treatment in children with severe acute asthma (SAA) is still a matter of debate. We assessed the efficacy of adding a salbutamol loading dose to continuous infusion with salbutamol in children admitted to a pediatric intensive care unit (PICU) with SAA. This multicentre, placebo-controlled randomized trial in the PICUs of four tertiary care children’s hospitals included children (2–18 years) with SAA admitted between 2017 and 2019. Children were randomized to receive either a loading dose IV salbutamol (15 mcg/kg, max. 750 mcg) or normal saline while on continuous salbutamol infusion. The primary outcome was the asthma score (Qureshi) 1 h after the intervention. Analysis of covariance models was used to evaluate sensitivity to change in asthma scores. Serum concentrations of salbutamol were obtained. Fifty-eight children were included (29 in the intervention group). Median baseline asthma score was 12 (IQR 10–13) in the intervention group and 11 (9–12) in the control group (*p* = 0.032). The asthma score 1 h after the intervention did not differ significantly between the groups (*p* = 0.508, β-coefficient = 0.283). The median increase in salbutamol plasma levels 10 min after the intervention was 13 μg/L (IQR 5–24) in the intervention group and 4 μg/L (IQR 0–7) in the control group (*p* = 0.001). Side effects were comparable between both groups.

*Conclusion*: We found no clinical benefit of adding a loading dose IV salbutamol to continuous infusion of salbutamol, in children admitted to the PICU with SAA. Clinically significant side effects from the loading dose were not encountered.

**Table:** 

**What is Known:** *• Pediatric asthma guidelines struggle with an evidence-based approach for the treatment of SAA beyond the initial steps of oxygen suppletion, repetitive administration of inhaled β2-agonists, and systemic steroids.* *• During an SAA episode, effective delivery of inhaled drugs is unpredictable due to severe airway obstruction.*	
**What is New:** *• This study found no beneficial effect of an additional loading dose IV salbutamol in children admitted to the PICU.* *• This study found no clinically significant side effects from the loading dose.*

**Supplementary Information:**

The online version contains supplementary material available at 10.1007/s00431-022-04576-8.

## Introduction

Severe acute asthma (SAA, status asthmaticus) is a severe or life-threatening asthma exacerbation that does not respond to oxygen supply, repetitive administration of inhaled β2-agonists, and systemic steroids. Pediatric asthma guidelines struggle with an evidence-based approach for the treatment of SAA beyond these initial steps [1–4]. During an SAA episode, effective delivery of inhaled drugs is unpredictable due to severe airway obstruction [5]. Therefore, intravenous (IV) salbutamol administration might be more effective. Still, data on the efficacy of this treatment strategy are lacking.

Salbutamol is a racemic mixture. The pharmacologic activity resides predominantly in the (R)-isomer. The elimination of (R)-salbutamol is much more rapid than that of (S)-salbutamol, which leads to higher plasma concentrations of (S)-salbutamol. There are concerns that high exposure to particularly S-salbutamol may have negative effects [6]. However, very little is known about the pharmacokinetics (PK) and pharmacodynamics (PD) of IV salbutamol in children and about the rationale behind current dosing strategies. Following current international guidelines [5, 7, 8], children receive much higher doses of continuous IV salbutamol per kilogram of weight than do adults. The PK-PD of IV salbutamol in children and adults appear to be similar, but data are limited [9]. A previous pilot study [6] on the PK of IV salbutamol in children has yielded a model of IV R- and S-salbutamol that described the data well and suggested a loading dose of salbutamol in children.

Studies in which children admitted to the emergency department (ED) received a single loading dose of salbutamol showed that this was associated with a reduction in length of hospital stay (12–28 h earlier discharge from the hospital) and lesser need of inhaled salbutamol maintenance [10–12]. Serious toxicity was not encountered. However, these studies had low sample sizes, used different outcomes and different asthma severity scores, and none described the relation between PK and PD. Furthermore, intervention studies in a PICU setting have not been performed. Another complicating factor is the lack of valid and reliable asthma severity scores.

The primary objective of our study was to assess the efficacy of an additional loading dose IV salbutamol in children admitted to a PICU with SAA, versus standard initiation of IV salbutamol.

## Materials and methods

Between April 2017 and June 2019, we prospectively identified children with SAA — defined as an acute asthma exacerbation that does not respond to conventional treatment with bronchodilators and systemic corticosteroids [13, 14] — who had been admitted to four of the seven PICUs in the Netherlands. All children with SAA between the ages of 2 and 18 years who did not respond to initial treatment and, therefore, had to receive continuous infusion of salbutamol according to the Dutch SAA guideline [15] were eligible. Exclusion criteria were (1) heart disease that interferes with normal asthma treatment, (2) pre-existing chronic pulmonary condition other than asthma (e.g. cystic fibrosis, bronchopulmonary dysplasia, bronchiolitis obliterans), (3) Down’s syndrome, (4) primary or secondary immunodeficiency, (5) having received a loading dose of IV salbutamol prior to study enrollment, (6) admitted more than 2 h before start of study, and (7) on invasive mechanical ventilation before receiving study medication or placebo (which would prevent assessment of the clinical asthma score). Current Dutch national guidelines [15] recommend PICU admission once a child has received IV salbutamol, regardless of the dosage.

**Fig. 1:**
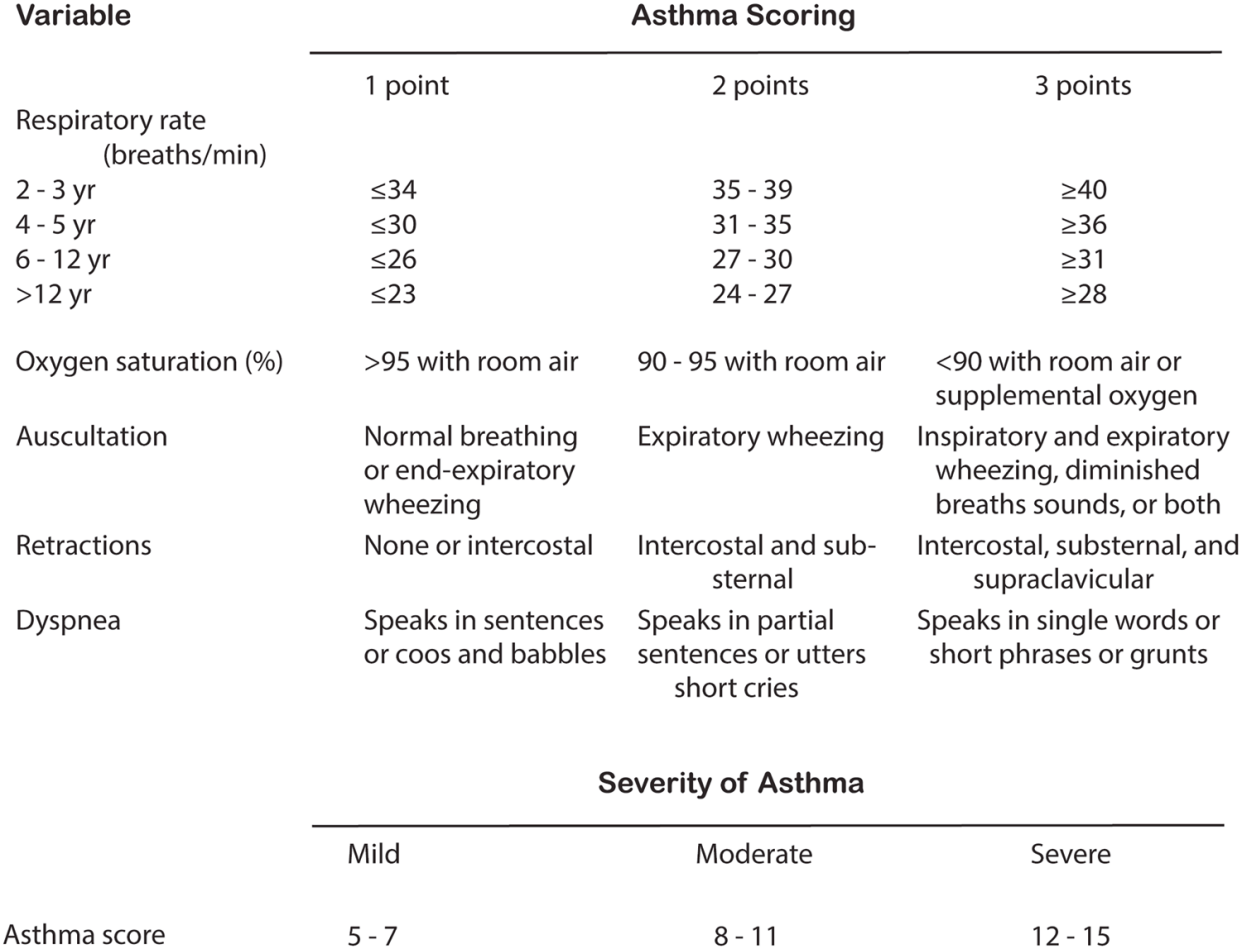
The asthma score (by Qureshi)

The effect of SAA treatment was determined from the asthma score developed by Qureshi and colleagues (Fig. 1) [16]. Although the most commonly used asthma severity scores show insufficient validity, the above-mentioned asthma score and the pediatric respiratory assessment measure (PRAM) [17] had the best test results regarding validity, reliability and utility [18, 19]. The asthma score is the most frequently used asthma severity score in the Netherlands. The main study endpoint was a difference in the participants’ asthma score of ≥ 2 points between the intervention and control groups 1 h after having received an IV salbutamol loading dose. Asthma scores were assessed prior to administration of the study medication and 10 min, 1, 3, 6, 12 and 24 h thereafter. Secondary outcomes were the maximum rate and duration of infusion of IV salbutamol, the total (cumulative) dose of IV salbutamol, length of PICU stay, need for other medication, need for (non-)invasive mechanical ventilation, the frequency of side effects, and serum concentrations of salbutamol.

Blocked randomization with randomly selected block sizes was applied, whereby blocks were stratified by centre. Study vials contained either salbutamol (500 mcg/ml) or placebo (sodium chloride 0.9%). The medication bolus (15 mcg/kg salbutamol with a maximum of 750 mcg) or placebo bolus was prepared according to the manufacturer’s instructions. The study medication was administered IV over a period of 10 min. In both groups, continuous infusion of salbutamol was started at the same time as the study medication or continued in children who already received salbutamol IV. The loading dose of 15 mcg/kg was based on international guidelines [5, 7] and a previous pilot study into the PK of IV salbutamol in children [6].

For the purpose of determining salbutamol serum concentrations, blood samples were drawn just before administration of the study medication or placebo, and 10 min, 1 h and 24 h (or prior to discharge) thereafter.

The Research Ethics Committee of the Erasmus University Medical Center Rotterdam (MEC 2016–402) approved the study and allowed either a priori or deferred informed consent.

See the online supplement for details on the randomization procedure, study medication, blood samples, and deferred consent.

### Sample size calculation

A sample size calculation using the ANCOVA model yields the following sample sizes: 17 patients for a power of 80% and 22 patients for a power of 90% (alpha = 0.05). Analysis of a database containing 5900 asthma scores recorded in the Erasmus MC – Sophia Children’s Hospital confirmed this estimate. This sample size calculation assumed a fixed effect of centre. We increased the sample size by 20% to compensate for potential missing data and/or dropouts. This yielded a final sample size of 56; i.e., 28 patients per group.

### Analyses

Data are presented as mean and standard deviation (SD) or median and interquartile range (IQR) if appropriate. Differences between groups were analyzed using *t*-tests for normally distributed variables, Mann–Whitney tests for continuous variables that were not normally distributed, and chi-square or Fisher’s exact tests were used to assess categorical variables. The linear-by-linear association chi-square test was used for ordinal categories. Analysis of covariance (ANCOVA) models were used to evaluate sensitivity to change in asthma scores after 1 and 6 h after the study medication, controlling for centre, baseline asthma score (before start study medication), and duration of IV salbutamol prior to study medication. Statistical analyses were carried out in SPSS version 25 (IBM Corp., Armonk, NY, USA) and a two-sided significance level of 0.05 was used.

## Results

**Fig. 2:**
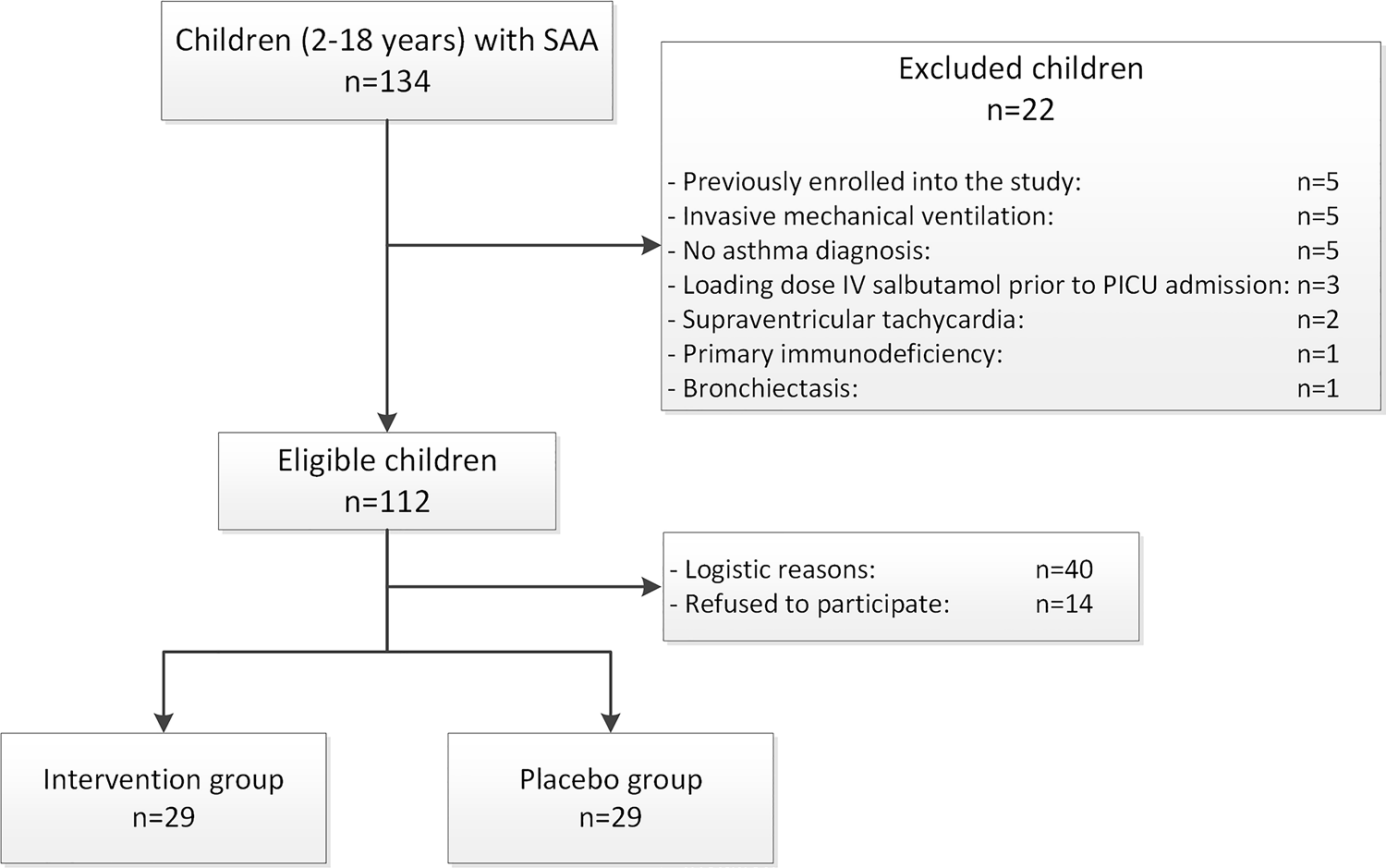
Flowchart inclusion

Fifty-eight children were included into the study (Fig. 2). Baseline characteristics are shown in Table 1. Before PICU admission, each child received continuous nebulization with salbutamol, prednisone and a bolus of magnesium sulphate (MgSO_4_) according to the Dutch national SAA guideline.

**Table 1:** Baseline characteristics

	**Intervention group (n = 29)**	**Control group (n = 29)**	***P* value**
Age in years	5 (3–9)	8 (5–13)	**.03**
2–4 years	12 (41)	6 (21)	.09
5–18 years	17 (59)	23 (79)	
Male gender	22 (76)	19 (66)	.39
Caucasian	12 (43)	15 (54)	.42
Allergic sensitization	15 (52)	14 (48)	.96
Reported smoke exposure	8 (28)	11 (38)	.23
Diagnosed with asthma prior to admission	24 (83)	23 (79)	.74
Prior SAA hospital admissions			
Non-PICU admission	16 (55)	17 (59)	.79
PICU admission	5 (17)	10 (35)	.13
Medication prior to PICU admission^a^			
Continuous nebulization salbutamol	29 (100)	29 (100)	
Prednisone	29 (100)	29 (100)	
Magnesium sulphate (MgSO_4_)	29 (100)	29 (100)	
Second bolus MgSO_4_	6 (21)	5 (18)	.74

Data are presented as median (IQR) or number (%)

^a^Medication administered at the emergency department or a general ward

### Primary endpoint

**Fig. 3:**
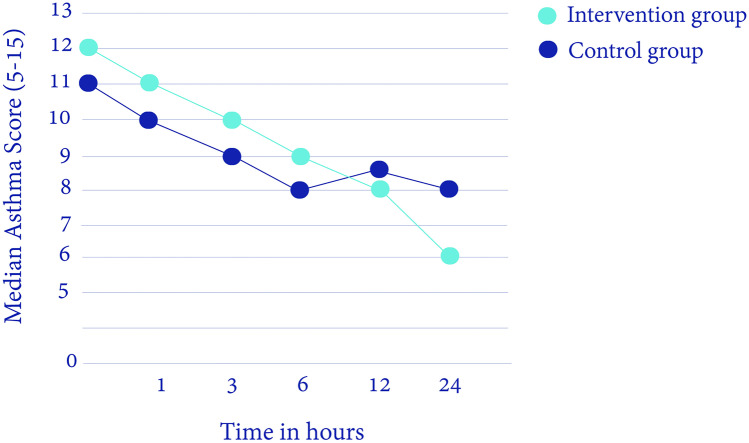
The median asthma score during PICU admission the first 24 h (Y-axis: median asthma score (5–15), X-axis: time in hours after PICU admission)

The time between start of IV salbutamol infusion and administration of study medication was > 100 min in both groups, with a median of 154 min (IQR 124–187) in the intervention group and a median of 108 min (IQR 75–158) in the control group (*p* = 0.043). The median baseline asthma score (at start of study medication) in the intervention group was 12 (10–13) versus 11 (9–12) in the control group (*p* = 0.03). The median asthma score 1 h after the intervention was 11 (9–12) versus 10 (8–11) in the control group (*p* = 0.06) (Table 2, Fig. 3). After correction for the baseline asthma score, centre, and duration of continuous infusion of IV salbutamol, there were no statistically significant differences between both groups in asthma score one and 6 h after administration of the study medication or placebo (Table 3). An additional analysis to correct for age did not show a significant effect of age on asthma score 1 h after the intervention (with a beta effect of 0.54 with a 95% CI of − 0.45/1.53 and a *p*-value of 0.28).

**Table 2:** PK/PD data

	**Intervention** **group (n = 29)**	**Control** **group (n = 29)**	***P*-value**
Asthma score at PICU admission	12 (11–13)	11 (10–12)	**.04**
Baseline asthma score^a^	12 (10–13)	11 (9–12)	**.03**
Asthma score 1 h after intervention	11 (9–12)	10 (8–11)	.06
Asthma score 6 h after intervention	9 (8–11)	8 (7–10)	.23
∆ Asthma score at baseline and 1 h after intervention	− .8 (1.6)	− 0.8 (1.4)	.89
∆ Asthma score at baseline and 6 h after intervention	− 2.0 (2.3)	− 1.7 (1.7)	.60
Maximum rate of salbutamol IV in mcg/kg/min	2 (0.1–10)	1.5 (0.3–6.0)	.69
Total cumulative dose of IV salbutamol in mcg/kg	3180 (751–6636)	1671 (706–5873)	.54
Duration of salbutamol IV in hours	43 (26–72)	31 (18–43)	.23
R-salbutamol baseline plasma level in μg/L, median (range)^b^	37 (7–191)	51 (6–219)	.21
S-salbutamol baseline plasma level in μg/L, median (range)^b^	65 (16–318)	79 (18–347)	.40
∆ R-salbutamol plasma level before and 10 min after intervention	13 (5–24)	4 (0–7)	**.001**
∆ R-salbutamol plasma level before and 1 h after intervention	16 (3–40)	11 (3–29)	.51
∆ S-salbutamol plasma level before and 10 min after intervention	18 (10–28)	4 (0–9)	** < .001**
∆ S-salbutamol plasma level before and 1 h after intervention	26 (6–48)	12 (1–33)	.17

Data are in median (IQR), mean (SD), ∆ = difference

^a^At start of the intervention

^b^Before administration of the study medication

**Table 3:** ANCOVA analysis

**Dependent var**	**Asthma score at 1 h**			**Asthma score at 6 h**		
**Parameter**	**β coefficient**^a^	**95% CI**	***p*-value**	***β* coefficient**^a^	**95% CI**	***p*-value**
Intercept	1.453	− 2.752/5.659	.490	2.137	− 3.282/7.555	.432
[Centre 1]	− .043	− 3.050/2.964	.977	2.425	− 1.459/6.309	.215
[Centre 2]	− .072	− 3.127/2.983	.962	1.098	− 2.848/5.045	.578
[Centre 3]	1.533	− 1.910/4.976	.375	2.690	− 1.750/7.130	.229
[Centre 4]	Reference	.	.	Reference	.	.
[Randomisation = Loading dose]	.283	− .570/1.136	.508	.237	− .876/1.350	.670
[Randomisation = Placebo]	Reference	.	.	Reference	.	.
Baseline asthma score	.757	.500/1.015	< 0.0005	.476	.144/.809	.006
Duration salbutamol infusion before study medication, in hours	.099	− .232/.431	.550	− .174	− .612/.263	.426

^a^β coefficients indicate how much a dependent variable changes per each unit variation of the independent variable, taking into account the effect of the other independent variables in the model. For categorical variables, β coefficients represent the effect of moving from the reference category (0) to another

### Secondary outcomes

Salbutamol plasma levels 10 min after administration of the loading dose compared to baseline had increased significantly more in the intervention group (Table 2). One hour after administration of the loading dose, there was no significant difference in salbutamol plasma levels between both groups.

Adjunct therapies and respiratory support were similar between both groups (Table 4). Hypotension (arterial blood pressure if available) was defined as a pressure less than the 5^th^ percentile of the systolic blood pressure [23], and was documented in 15 patients: 11 in the control group, and 4 in the intervention group (*p* = 0.077). None required inotropic support. One patient in the placebo group suffered from supraventricular tachycardia, and had recovered without complications after the IV salbutamol was discontinued. In the intervention group, 29 patients developed hyperglycemia (> 8 mmol/L), versus 27 patients in the control group (*p* = 0.150). None of the patients had been treated with insulin [20]. Tachycardia was present in all but one patient during PICU admission.

**Table 4:** Presentation and PICU management

	**Intervention group (n = 29)**	**Control group (n = 29)**		***P*-value**
pH at PICU admission, mean (SD)	7.37 (0.1)	7.37 (0.1)	.956	
PCO_2_ at PICU admission (kPa), mean (SD)	5.4 (1.6)	5.3 (1.3)	.747	
Asthma severity score (Qureshi), n (%)			**.007**	
Mild (5–7)	0 (-)	1 (3)		
Moderate (8–11)	10 (35)	19 (66)		
Severe (12–15)	19 (66)	9 (31)		
Adjunct therapies, n (%)			.706	
Ketamine	4 (14)	0 (-)		
Sodium Bicarbonate	1 (3)	1 (3)		
Theophylline	0 (-)	1 (3)		
DNAse	1 (-)	1 (-)		
Maximal respiratory support, n (%)			.753	
None	0 (-)	1 (3)		
Nasal cannula	4 (14)	3 (10)		
Non-rebreathing mask	11 (38)	10 (35)		
High-flow nasal cannula	14 (48)	14 (48)		
Non-invasive ventilation	0 (-)	0 (-)		
Invasive mechanical ventilation^a^	0 (-)	1 (3)		
PICU length of stay in hours, mean (SD)	58 (28)	55 (36)	.696	

^a^Invasive mechanical ventilation after administration of the study medication, since invasive mechanical ventilation was an exclusion criteria

## Discussion

In this randomized, multicentre, placebo-controlled trial, no benefit was found of the administration of an adjuvant loading dose of IV salbutamol in children admitted to a PICU with SAA (most of whom were already on IV salbutamol infusion), when comparing the clinical asthma score, co-medication, respiratory support, and PICU length of stay with those of controls who had received normal saline. The administration of an adjuvant loading dose of IV salbutamol was not associated with side effects.

Our findings are only partly consistent with those of the previous studies. As early as 1984, Bohn and colleagues had suggested that a loading dose of IV salbutamol might be efficacious. They demonstrated a decrease of PaCO_2_ in 11 of 16 children with SAA patients after a loading dose of 10 mcg/kg followed by continuous infusion of salbutamol [21]. This study is not comparable with the present study (e.g., different loading dose and outcome variable).

Single (small) centre randomized studies performed in the 1990s in the ED showed shorter recovery time (e.g., cessation of inhaled medication) in children with SAA who had received a bolus of IV salbutamol (15 mcg/kg in 10 min), while no side effects were reported [10, 11]. In contrast to the present study, a loading dose of salbutamol was not followed by or added to continuous salbutamol infusion.

In 2007, Bogie and colleagues performed a randomized, double blind, placebo-controlled trial in children presenting to an ED with SAA. Patients were randomized to receive either IV terbutaline (a loading dose followed by continuous infusion) or IV normal saline while on continuous high-dose nebulized albuterol. Outcome measures revealed a trend toward clinical improvement in the terbutaline group [12]. In the present study, we found no clinical benefit of a loading dose of salbutamol in children who were already on continuous infusion with salbutamol, in a PICU setting. None of the earlier studies described PK data [10, 11].

### How can our findings — e.g., lack of efficacy of a loading dose IV salbutamol — be explained?

The pharmacologic activity of salbutamol resides predominantly in the (R)-isomer, with little or no activity, and concerns about adverse reactions, attributed to the (S)-isomer [6, 22]. Based on a previous population PK model of IV R- and S-salbutamol in children with SAA, we considered that administering a loading dose might be efficacious to reach higher initial R-salbutamol concentrations with a possible therapeutic advantage [6]. All study participants had received nebulized salbutamol before IV administration, which intervention as such also leads to elevated plasma levels of salbutamol [23]. Furthermore, the majority were already on IV salbutamol infusion (median dose at baseline of 0.5 mcg/kg/min) before inclusion. Although administration of a salbutamol-loading dose resulted in significantly higher plasma levels 10 min after administration of the loading dose, this effect did not remain significant after 1 h. Treatment with IV salbutamol resulted in high inter-individual differences in plasma salbutamol levels, with no clear correlation with pharmacodynamic parameters (e.g., asthma score and heart rate). Based on our data and our previous PK model of R- and S-salbutamol [6], we can safely conclude that a steady state was reached 1 h after start of continuous infusion of salbutamol.

Our study does not exclude a possible benefit of a loading dose of IV salbutamol in children with severe or near-fatal SAA in a pre-hospital setting or ED with a very low baseline concentration of salbutamol, as previously illustrated in a case report [24].

There is a large variation in SAA treatment worldwide, and many pharmacological interventions are being applied [4, 25]. Furthermore, guideline recommendations represent considerable variation in the management of asthma exacerbations, affecting diagnostic and treatment decisions [25]. However, evidence indicates that the Dutch national SAA guideline is well adhered to in our country [14]. Therefore, we hold that the starting point for our patients in this study is similar.

In SAA treatment, bronchodilation is the goal of salbutamol as a selective β2-adrenoreceptor agonist with potent smooth muscle relaxant properties. Could it be the case that inflammation or mucus plugging are more predominant in the cause of severe airflow obstruction in pediatric SAA? To be able to individualize treatment, we need to gain better understanding of the pathophysiology or “clinical phenotype” of SAA children.

## Limitations/strengths

Strengths of the present study include the randomized placebo-controlled design, the PK-PD analysis, and the participation of the majority of Dutch PICUs. Still, some limitations need to be addressed. First, most children had received continuous infusion with salbutamol for more than 1 h before start of study medication or placebo. Unfortunately, in regional hospitals, it was not feasible to administer a loading dose of IV salbutamol (before the continuous infusion of IV salbutamol). Second, although the asthma score by Qureshi and colleagues is the best available asthma severity score, there is need for a better scoring instrument for clinical and research reasons. To our knowledge, there have never been subsequent studies validating this score for different populations. Moreover, the precise characteristics of this score have not yet been determined; the original article mentions an interrater reliability of 80%. Thus, it seems that a better scoring instrument is needed for clinical and research settings. However, since also co-medication, respiratory support and PICU length of stay did not differ between the intervention group and the control group, we may assume that there was indeed no difference in the course of the SAA episode between the groups.

## Conclusion

In this multicentre, placebo-controlled randomized trial, we found no beneficial effect of a loading dose of IV salbutamol in SAA children admitted to a PICU (majority already on IV salbutamol infusion) with regard to clinical asthma score, co-medication, respiratory support and PICU LOS. Nor were there significant side effects. Future studies should focus on the efficacy of a loading dose of IV salbutamol in the ED, before or simultaneous with the start of continuous salbutamol infusion, with the ultimate goal of preventing further deterioration of respiratory distress (and PICU admission). Lastly, a validated asthma score is needed to study the efficacy of different interventions in the context of ED and PICU care.

## Supplementary Information

Below is the link to the electronic supplementary material.

Supplementary file1 (DOCX 16 KB)

## Data Availability

The authors confirm that the data supporting the findings of this study are available within the article and its supplementary materials.

## Abbreviations

*ANCOVA*: Analysis of covariance
*ED*: Emergency department
*IV*: Intravenous
*IQR*: Interquartile range
*LOS*: Length of stay
*PD*: Pharmacodynamics
*PICU*: Pediatric intensive care unit
*PK*: Pharmacokinetics
*SAA*: Severe acute asthma
*SD*: Standard deviation

## Code availability

SPSS version 25 (IBM Corp., Armonk, NY, USA).

## Declarations

### Trial register

clinicaltrials.gov (NCT03493503).
