# Supplementary material for: Efficacy of a loading dose of IV salbutamol in children with severe acute asthma admitted to a PICU: a randomized controlled trial
Source: Eur J Pediatr. 2022 Aug 3;181(10):3701–9. doi: 10.1007/s00431-022-04576-8 (PMC9508206; doi:10.1007/s00431-022-04576-8)
Supplement: Supplementary file 1 — Supplementary file1 (DOCX 16 KB) [file 431_2022_4576_MOESM1_ESM.docx]

**Efficacy of a loading dose of IV salbutamol in children with severe acute asthma admitted to a PICU: a randomized controlled trial**

Shelley A. Boeschoten, MD^1^, Corinne M. Buysse, MD, PhD^1^, Brenda C. de Winter, MD, PhD^2^, Joost van Rosmalen, MD, PhD^3^, Johan C. de Jongste, MD, PhD^4^, Rogier C. de Jonge, MD, PhD^1^, Sabien G. Heisterkamp, MD^5^, Job B. van Woensel, MD, PhD^5^, Martin C. Kneyber, MD, PhD^6^, Annelies van Zwol, MD, PhD^7^, Annemie L. Boehmer, MD, PhD^8,9^, Matthijs de Hoog, MD, PhD^1^, on behalf of the Dutch collaborative PICU research network (SKIC) * A complete list of research network participants is provided in the acknowledgements

**Online Data Supplement**

**Methods**

We prospectively identified children with SAA admitted to 4 Dutch academic PICUs. All children (2-18 years of age) admitted for SAA between April 2017 and June 2019 with the need for continuous infusion of IV salbutamol were eligible. Exclusion criteria were as follows: (1) heart disease that interferes with normal asthma treatment, (2) underlying respiratory illness other than asthma, (3) Down’s syndrome, (4) primary or secondary immunodeficiency, (5) a loading dose of IV salbutamol prior to study enrollment, (6) admission to a PICU for >2 hours, and (7) patients intubated before receiving study medication (no assessment of clinical asthma score possible). The asthma score was a subjective description for determining asthma severity and change in clinical condition, no absolute number was required to determine enrollment. Treatment levels before admission were determined according to the GINA guideline (1).

The effect of SAA treatment was determined by using the “Asthma Score” [Figure 1], as developed by Qureshi (2). To our knowledge there have never been subsequent studies validating this score for different populations. As such, the precise characteristics of this score have not been determined; the original article only mentions an interrater reliability of 80%. Asthma scores were assessed prior to administration of the study medication (baseline asthma score) and 10 minutes, 1, 3, 6, 12 and 24 hours after administration of the loading dose.

Randomisation was done using blocked randomisation with randomly selected block sizes, provided by the department of biostatistics of the Erasmus Medical Center Rotterdam. Blocks were stratified by center. The hospital pharmacy was responsible to produce sealed boxes with the correct contents. Study vials containing either salbutamol (500 mcg/ml) or placebo (sodium chloride 0.9%) were prepared. A blinded physician, (research) nurse or researcher retrieved a blinded box from a locked room in the participating center’s pharmacy. A non-blinded nurse, other than the treating (blinded) nurse, prepared the medication bolus (15 mcg/kg salbutamol with a maximum of 750 mcg) or placebo bolus (identical volume of NaCl 0.9% solution) according to the instructions included in the box. Next, the double-blind label was added to the prepared study medication/placebo. To optimize blinding the non-blinded individuals were instructed not to give any information about the preparation of medication to the treating nurse, physician or researcher. Once prepared, the study medication was administered via a syringe pump intravenously over a period of 10 minutes. The cannula was flushed with 5–10 mL saline after the saline or salbutamol was administered and then it was capped. Continuous infusion of salbutamol was started at the same time or continued in patients who already received salbutamol IV in the referring hospital, in order to ensure that both groups actually received treatment. Titration of the salbutamol dose was performed according to our national protocol, based on the clinical condition of the patient. The loading dose of 15 mcg/kg is based on international guidelines (3, 4) and a previous pilot study into the PK of IV salbutamol in children by our group (5). In this study a NONMEM model predicting PK of salbutamol in children shows that a loading dose of 15 mcg/kg results in an increase of serum concentration of 20-30 ng/mL, assuming a baseline concentration after nebulization of 20 ng/ml.

Before administration of the study medication blood was obtained to assess the current condition of the patient (blood gases) and for salbutamol serum concentration at baseline. Furthermore, 500 microliters (min. 150 microliters) of blood was drawn to determine salbutamol serum concentrations 10 minutes, 1 hour and 24 hours (or prior to discharge) after administration of the study medication. Blood samples for serum concentration of salbutamol were (where possible) taken concomitantly with samples obtained for standard care; either via indwelling arterial catheter (only if already in place for treatment purposes), venipuncture or finger prick. These plasma samples were analyzed for R- and S-salbutamol concentrations separately using a validated liquid chromatography–tandem mass spectrometry (LC-MS/MS) method; the limit of quantification of the method was 1.0 µg/L, with a linearity of between 1.0 and 500 µg/L for both R- and S-salbutamol (5).

The national guidelines for PICU admission did not change during the study period and included respiratory failure and imminent exhaustion, no clinical benefit after continuous nebulization of a bronchodilator and an IV bolus of magnesium sulphate, and the need for intravenous (IV) administration of salbutamol (which is an indication for PICU admission according to Dutch guidelines(6)), and/or the need for mechanical ventilation.

The Research Ethics Committee of the Erasmus Medical Center Rotterdam (MEC 2016-402) approved the study and allowed either a priori or deferred informed consent.

**Sample size calculation**

For this study the primary outcome was asthma score at 1 hour after administration of the intervention, corrected for baseline asthma score. The standard deviation for asthma score (based on a local database with ~5900 asthma scores) is 2.35. We assumed a correlation of 50% between asthma score at 1 hour after intervention and asthma score at baseline. Finally, we expected a difference of 2 points in asthma score between intervention and control

at 1 hour after intervention. A sample size calculation using the ANCOVA model yields the following sample sizes: 17 patients for a power of 80% and 22 patients for a power of 90%. Analysis of a database containing 5900 asthma scores recorded in the Erasmus MC – Sophia Children’s Hospital confirmed this estimate. This sample size calculation assumed a fixed effect of center. We increased the sample size by 20% to compensate for potential missing data and/or dropouts. This yields a final sample size of 28 patients per group, a total of 56.

**Analyses**

Data were presented as mean and standard deviation (SD) or median and interquartile range (IQR) if appropriate. Differences between groups were analyzed using t-tests for normally distributed variables, Mann-Whitney tests for continuous variables that were not normally distributed, and chi-square or Fisher’s exact tests were used to assess categorical variables. The linear-by-linear association chi-square test was used for ordinal categories. Analysis of covariance (ANCOVA) models were used to evaluate sensitivity to change in asthma scores after 1 and 6 hours after the study medication, controlling for center, baseline asthma score (before start study medication), and duration of IV salbutamol prior to study medication. Statistical analyses were carried out in SPSS version 25 (IBM Corp., Armonk, NY, USA) and a two-sided significance level of 0.05 was used.

**Deferred consent**

For this study we used deferred consent. Both patients and caregivers found themselves in an acute and overwhelming situation. They had to process a lot of information in a short time. This understandably leads to stress. Asking for consent for a scientific study adds even more information and pressure. As such, asking for consent in this situation is not ethically valid. Since SAA is a life-threatening situation that requires rapid or immediate treatment, this does not leave sufficient time for a proper informed consent procedure prior to the loading dose intervention. Parents did not have the time to give informed consent after thoughtful consideration of the patient information form. In the Netherlands, informed consent for minors must be signed by both caregivers, as well as the patient if aged ≥12 yrs. The fact that patients are transported to our hospital by ambulance accompanied by (at maximum) 1 parent, or due to a divorce, etc. it is highly unlikely that the 2nd caregiver can be contacted and present before start of the intervention.

**References**

E1. Global Initiative for Asthma. Available at: https://www.brit-thoracic.org.uk/quality-improvement/guidelines/asthma/. Accessed 2019

E2. Qureshi F, Pestian J, Davis P, et al: Effect of nebulized ipratropium on the hospitalization rates of children with asthma. *N Engl J Med* 1998; 339: 1030-1035.

E3. BTS/SIGN British guideline on the management of asthma. Available at: https://www.brit-thoracic.org.uk/quality-improvement/guidelines/asthma/. Accessed July 2019

E4. The Australian Asthma Handbook. Available at: https://www.asthmahandbook.org.au/acute-asthma/clinical/add-on-treatment. Accessed September 2020

E5. Vet NJ, de Winter BCM, Koninckx M, et al: Population Pharmacokinetics of Intravenous Salbutamol in Children with Refractory Status Asthmaticus. *Clin Pharmacokinet* 2019.

E6. Dutch guideline for the management of acute asthma in children. Available at: https://www.nvk.nl/Portals/0/richtlijnen/acuut%20astma/Methodenacuutastma.pdf. Accessed 2012
